# Ventricular Repolarization and Calcium Transient Show Resonant Behavior under Oscillatory Pacing Rate

**DOI:** 10.3390/biom12070873

**Published:** 2022-06-23

**Authors:** Massimiliano Zaniboni

**Affiliations:** Department of Chemistry, Life Sciences and Environmental Sustainability, University of Parma, Parco Area delle Scienze 11/A, 43124 Parma, Italy; massimiliano.zaniboni@unipr.it

**Keywords:** cardiac EC coupling, cardiac action potential, cardiac numerical modeling, cardiac repolarization, variability of cardiac pacing

## Abstract

Cardiac EC coupling is triggered by rhythmic depolarizing current fronts originating from the sino-atrial node, and the way variability in rhythm is associated with variability in action potential duration (APD) and, in turn, in the variability of calcium transient amplitude (CTA) and contraction is a key determinant of beating stability. Sinusoidal-varying pacing rate is adopted here in order to establish whether APD and CTA oscillations, elicited in a human ventricular AP model (OR) under oscillatory pacing, are consistent with the dynamics of two coupled harmonic oscillators, e.g., a two-degree-of-freedom system of mass and springs (MS model). I show evidence that this is the case, and that the MS model, preliminarily fitted to OR behavior, retains key features of the physiological system, such as the dependence of APD and CTA oscillation amplitudes from average value and from beat-to-beat changes in pacing rate, and the phase relationship between them. The bi-directionality of coupling between APD and CTA makes it difficult to discriminate which one leads EC coupling dynamics under variable pacing. The MS model suggests that the calcium cycling, with its greater inertia chiefly determined by the SR calcium release, is the leading mechanism. I propose the present approach to also be relevant at the whole organ level, where the need of compact representations of electromechanical interaction, particularly in clinical practice, remains urgent.

## 1. Introduction

Periodic behavior appears in a variety of biological contexts covering a 10^10^-wide range of oscillating frequencies [1,2], and cardiac cellular excitation–contraction (EC) coupling is a paradigmatic example. EC coupling consists of non-linear oscillations of membrane potential that trigger and control oscillations in intracellular calcium concentration (calcium transient CT) and, in turn, in cell shortening, finally resulting in the rhythmic contraction of the heart [3]. For the working myocardium, the oscillating system can be seen as externally driven by the periodic injection of electrotonic depolarizing current pulses originally initiated from the sinus node and following each other at an approximately constant *sinus* rate. The cycle length (CL) of sinus rhythm though is always associated with beat-to-beat variability (heart rate variability HRV), which is partly intrinsic, due to stochastic changes in the clock mechanisms underlying diastolic depolarization [4], partly associated with higher level regulations, such as autonomic modulation, circadian rhythms, baroreflex, and others [5]. Some of these beat-to-beat CL changes are random, some are linearly developing, some single events, other periodic. Cardiac physiologists are interested in the way CL changes affect the time course of membrane repolarization, since it is action potential duration (APD), or the QT interval of ECG at body surface level, that determine, via EC coupling, the partition of the cardiac cycle into systole and diastole [6]. The relationship between changes in CL and changes in repolarization goes under different denominations, depending on the type of CL changes: rate dependence (RD) for stationary changes, electrical restitution (ER) for sudden changes from stationary conditions, and beat-to-beat ER for sudden changes under dynamic conditions [6,7,8,9,10]. As we use rate dependence and restitution for APD (or QT), we can similarly refer to the same properties for the amplitude of CT oscillations (CTA), or their organ counterpart in the systolic pressure developed by the heart chambers. We should bear in mind however that APD and CTA are bi-directionally coupled, i.e., AP triggers CT, which in turn feeds back into AP dynamics at each beat [11,12]. Beat-to-beat control of CL over both APD and CTA, thus, is described by restitution properties, but when and whether it is the restitution of APD that controls that of CTA or vice versa, is still a matter of debate [13]. This becomes relevant, for example, for discriminating between electrically or calcium-driven repolarization abnormalities, such as APD alternans, which initiate arrhythmias. Experimental and theoretical studies have provided, at the cellular, tissue, and organ level, rules that regulate electrical and calcium restitution in the heart [14], and transfer functions have also been provided that turn one APD and the corresponding CT into the following in the sequence, when CL changes over time [15]. However, despite these efforts, the question of mutual interaction remains largely unanswered.

The present study is based on numerical simulations on the O’Hara et al. 2011 (OR) model of human ventricular AP [16] under oscillatory pacing, also in comparison with other numerical models. I move from the observation that when pacing CL oscillates, APD and CTA also do. Despite these experimental observables originating from non-linear oscillations of membrane potential (the AP) and intracellular calcium concentration (the CT), for a broad range of sinusoidal pacing CL parameters (basic CL, interval of CL variability, and rate of CL changes), APD and CTA behave similar to coupled harmonic oscillators. This makes it possible to describe key features of their dynamics in terms of a simple mechanical model.

Oscillations in pacing rate have been observed in heart failure, post-myocardial infarction, and hypertension, and frequently precede the onset of arrhythmias [17]. Additionally, sinusoidally varying heart rate patterns have been recorded in newborn infants [18] and applied through artificial pacemakers in heart failure patients in order to dynamically manipulate their respiratory cycle [19]. Despite their significance in several pathophysiological states, harmonic oscillations in pacing CL are not adopted here to reproduce any of these conditions. Rather they are used here as a tool to investigate and re-think the dynamics of EC coupling in terms of a very simple two-degree-of-freedom system of two masses and three springs and dampers (MS model), where one of the masses is externally accelerated with a sinusoidally varying force. In addition, oscillatory pacing makes it possible to control and continuously change the average and the variability range of pacing parameters we are interested in. Of course there is no physical meaning in the mechanical nature of the chosen MS model other than its dynamics, which is so frequently adopted in the interpretation of oscillating natural phenomena and is applied here for the first time to the study of the cardiac EC coupling.

The MS model reproduces the steady state and beat-to-beat rate dependence of EC coupling as simulated in the OR model, as well as the phase relationship between the electrical and calcium counterparts. It also suggests that the dynamics of calcium cycling overwhelms that of APD under oscillatory pacing, which draws new light into EC coupling dynamics.

## 2. Materials and Methods

All simulations presented in this study have been performed on OR human ventricular action potential (AP) model [16]. The CellML format of the model [20] was recompiled in its MATLAB version by means of COR facility at https://models.physiomeproject.org/electrophysiology (accessed on 20 June 2022). The ‘ode15s’ solver built into the R2020a version of MATLAB (The Math-Works, Inc., Natick, MA, USA) was used to integrate model equations. All simulations were run on a PC with Intel(R) Core (TM) i7, 2.8 GHz CPU. APs were elicited by simulating 0.5 ms-long current injections with an amplitude 50% above current threshold. AP duration was measured as APD −60 mV, i.e., the time between the maximum first derivative of membrane potential (V_m_) during the initial fast depolarization phase and the time during repolarization when V_m_ reached the value of −60 mV. Intracellular calcium concentration is one of the integrated variables of the OR model and the amplitude of its oscillations (CTA) is measured, for each beat, as the maximum value reached by [Ca^2+^]_i_ subtracted by its diastolic value right before the stimulation. For model comparison, simulations have been performed on two additional human ventricular models, the Ten Tusscher and Panfilov 2006 (TP) [21] and the Iyer–Mazhari–Winslow 2004 (IMW) [7].

Beat-to-beat electrical restitution (btb-ER) is defined in this study for AP sequences paced at variable pacing rate, as APD of each beat versus the preceding CL. It is also similar for CTA [22]. AP sequences under sinusoidally varying pacing (SVP) were obtained by making CL varying as follows:

(1) $$\mathrm{CL}\left(t_{N}\right)=\mathrm{BCL}+\sigma .\;\sin\left(\omega .t_{N}\right)$$

where BCL is the basic cycle length around which CL oscillates, *σ* is the half-range of CL oscillations, *ω* the angular frequency, and t*_N_* is the time at the onset of each stimulus current injection, thus:

(2) $$t_{N}=\sum_{i=1}^{N}{\mathrm{CL}}_{i}\;\;\;\;\;\;\;\;\;\;\;\;\;\;\;\;\;\;\;\;\;\;\;\;\;\;\;\;\;\;$$

and the period for an entire CL oscillation is:

(3) $$T=\frac{2\pi }{\omega }$$

### 2.1. Upper Limit for Beat-to-Beat CL Changes

If we consider the discrete series of CL values of Equation (1), the maximum value of the difference between consecutive CL values (ΔCL_max_) is the entire range of oscillation, i.e., 2σ, which corresponds to a period of 2*BCL and an ω value (ω_max_) of

ω_max_ = π/BCL (4)

Accordingly, ΔCL increases as ω does up to ω_max_, and decreases again when ω > ω_max_. The discrete nature of the series in (1) therefore sets the limit (ω < ω_max_) for which increasing ω makes beat-to-beat CL changes to increase. Since the maximum pacing rate (i.e., minimum BCL) that does not lead to APD alternans in the models under study is 300 (OR), 350 (TP), and 375 (IMW) ms, it follows that ω_max_, when BCL is expressed in s, is 10.5, 8.9, and 8.4 in the three, respectively. For the sake of brevity, I will call the range of allowed ω values ω-ROI (range of interest).

### 2.2. The Mass-Spring (MS) Model

In this study, I refer to two classical mechanical systems in order to idealize the forced oscillations of APD and CTA during SVP. Details on the dynamics of these type of systems are covered in textbooks on mechanical vibrations, to which I refer for the theory and for the MATLAB numerical and analytical solutions [23].

The first is a one-degree-of-freedom system, made of a single point mass (m) connected by a spring (stiffness k) and a damper (damping constant c) to a fixed wall and subjected to a force F along the unique dimension x (see schemes in the results section). The natural frequency of the oscillation of this system is:

(5) $$\omega_{0}={\left(\frac{k}{m}\right)}^{\frac{1}{2}}$$

When an oscillatory force

(6) $$F\left(t\right)={\;F}_{\mathrm{const}}+F_{\mathrm{osc}}\;\sin\left(\omega \;t\right)$$

is applied to m, the amplitude of oscillations increases as ω approaches ω_0_ (resonant frequency); the constant c modifies the phase relationship between F(t) and x(t) and the width of the resonance profile (the larger c, the broader the resonance peak). I will refer to this system as the M_i_S model.

The second is a two-degree-of-freedom system, made of two masses connected through 3 springs and 3 dampers (see schemes in the results section). I will refer to it as the MS model. The application of the Newton’s second law of motion to each of the masses gives the equation of motion for free vibrations (F(t) = 0):

(7) {m1d2x1dt2+(c1+ c2) dx1dt−c2dx2dt+(k1+ k2) x1−k2x2=0 m2d2x2dt2−c2dx1dt+(c2+ c3) dx2dt−k2x1+(k2+ k3) x2=0 

which can be studied for steady state solutions x_i_ of the type:

(8) $$x_{i}={\;x}_{\mathrm{is}}\;\sin\omega t+{\;x}_{\mathrm{ic}}\;\cos\omega t\;$$

where x_is_ is the amplitude vector of the sine component and x_ic_ is the cosine component of the solutions.

The matrix form of (7) is then:

(9) $$\left[\begin{array}{cc} -\omega^{2}\left[M\right]+\left[K\right] & \omega \;\left[C\right]\\ -\omega \;\left[C\right] & -\omega \;\left[M\right]+\left[K\right] \end{array}\right]\;\left\{\begin{array}{c} x_{\mathrm{ic}}\\ x_{\mathrm{is}} \end{array}\right\}=0\;$$

where i = 1,2, and:

(10) $$M=\left[\begin{array}{cc} m_{1} & 0\\ 0 & m_{2} \end{array}\right];\;C=\left[\begin{array}{cc} c_{1}+c_{2} & -c_{2}\\ -c_{2} & c_{2}+{\;c}_{3} \end{array}\right];\;K=\left[\begin{array}{cc} k_{1}+k_{2} & -k_{2}\\ -k_{2} & k_{2}+{\;k}_{3} \end{array}\right]$$

For non-trivial steady state solutions of (9), the determinant of the coefficients must be zero:

(11) $$\left|\begin{array}{cc} -\omega^{2}\left[M\right]+\left[K\right] & \omega \;\left[C\right]\\ -\omega \;\left[C\right] & -\omega \;\left[M\right]+\left[K\right] \end{array}\right|=0$$

which is the characteristic equation of system (9) and provides its normal modes of vibration ω_1_ and ω_2_. When an external periodic force of frequency ω (Equation (6)) is applied to m_1_, m_2_, or both, steady state oscillating solutions will be given by Equation (9), where the term on the right size will be the external forces instead of zero. Resonance will develop any time the frequency ω of the external force *F* approaches ω_1_ or ω_2_, and will make the amplitude of the oscillations of m_1_ and m_2_ (Δx_1_ and Δx_2_) increase.

## 3. Results

### 3.1. Periodic Pacing

**Figure 1:**
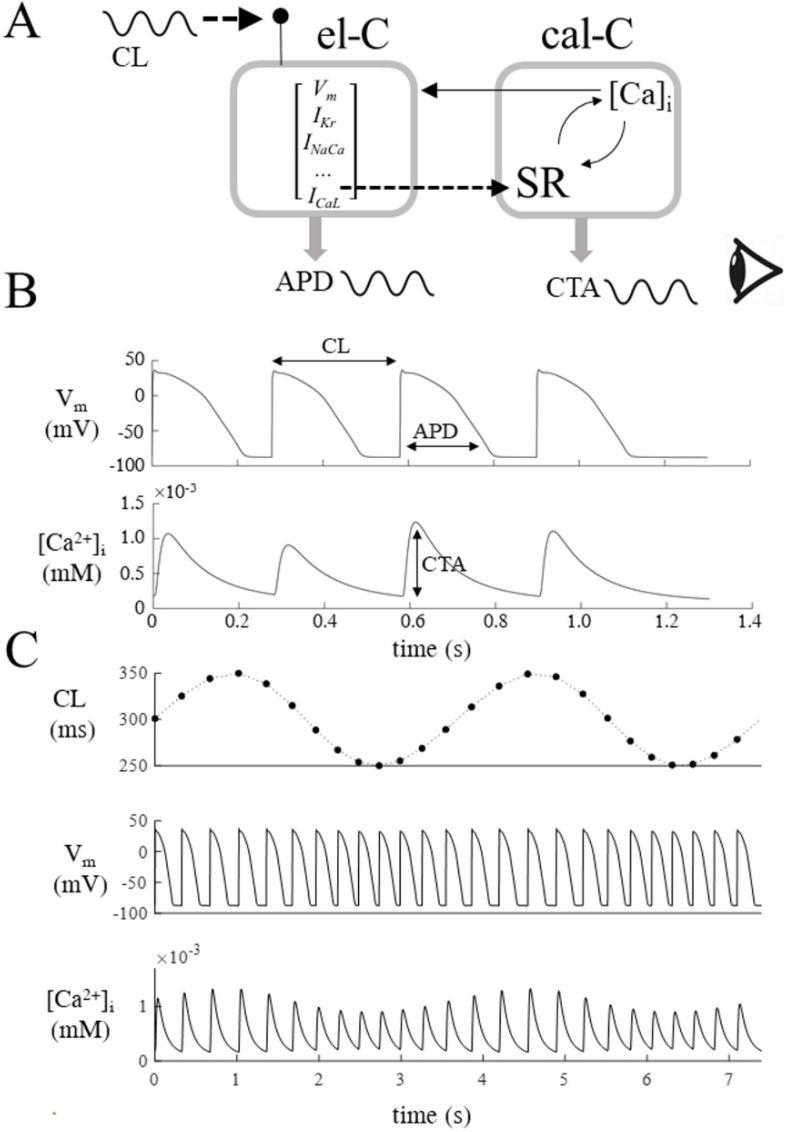
**Oscillations of APD and CTA under oscillatory pacing.** (**A**): Schematic representation of cellular ventricular EC coupling. The membrane excitability (el–C) and calcium handling (cal–C) counterparts are monitored experimentally through APD and CTA. el–C is paced at every CL via a brief current injection (I_st_) and, at the same CL, the calcium current I_CaL_ triggers cal–C to initiate the calcium cycle. (**B**): Sequences of APs and CTs are simulated while CL changes on a beat-to-beat basis, and corresponding APDs and CTAs are recorded. (**C**): When CL oscillates around a given value (top panel), APDs (middle pannel) and CTAs (bottom panel) also oscillate at the same frequency.

Figure 1A shows a schematic representation of EC coupling as it is modeled by OR model’s equations. Membrane electrical excitability is triggered by 0.5 ms-long constant current injections (I_st_) delivered every cycle length (CL). It is described, for each beat, by a set of time-varying state variables determining transmembrane ion fluxes, one of which, the L-type calcium current I_CaL_, triggers the sarcoplasmic reticulum (SR) calcium release and initiates contraction. I will label for brevity the electrically and I_CaL_-driven compartments as el–C and cal–C, respectively. Cytoplasmic [Ca^2+^]_i_ level is restored within every beat, while its excursion (the calcium transient, CT) feeds back into el–C through a number of mechanisms. Among the many dependent variables in play, the duration of the V_m_ excursion (APD) (top panel B) and the amplitude of the CT (CTA) (bottom panel B) are monitored over each cycle.

### 3.2. Rate (BCL) Dependence of Oscillations

**Figure 2:**
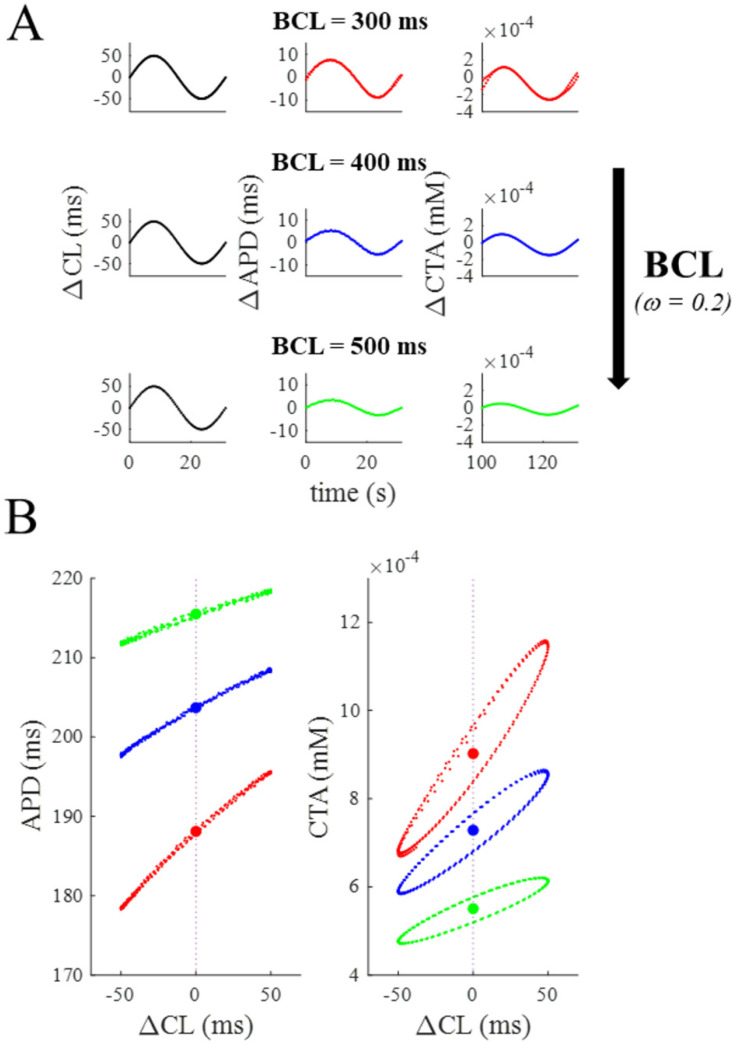
**Dependence of APD and CTA oscillations from BCL**. (**A**): When the OR model was paced according to Equation (1) with a low *ω* value and σ = 50 ms (first column), the amplitude of APD and CTA oscillations decreased with pacing frequency (1/BCL, values reported in figure) (black arrow). (**B**): btb-ER representations of the APD and CTA sequences of panel A with the same color code.

As I have shown previously [22,24,25], when ventricular AP models are paced with oscillating CL (Equation (1)), APDs and CTAs also oscillate (Figure 1C). Specifically (Figure 2), when CL oscillates within ±50 ms (σ) around a central value with a slow rate of beat-to-beat CL changes (ω = 0.2, an entire ΔCL cycle takes ~31 s), the amplitude of APD and CTA oscillations is pacing rate dependent, i.e., it decreases as the pacing rate (1/BCL) does (BCL = 500, 400, 300 ms; top to bottom in panel A). This can also be appreciated in the btb-ER representations of panel B. The average value of APD and CTA at the different BCLs (colored dots in figure) represents what is usually called rate dependence of these parameters.

### 3.3. Frequency (ω) Dependence of Oscillations

**Figure 3:**
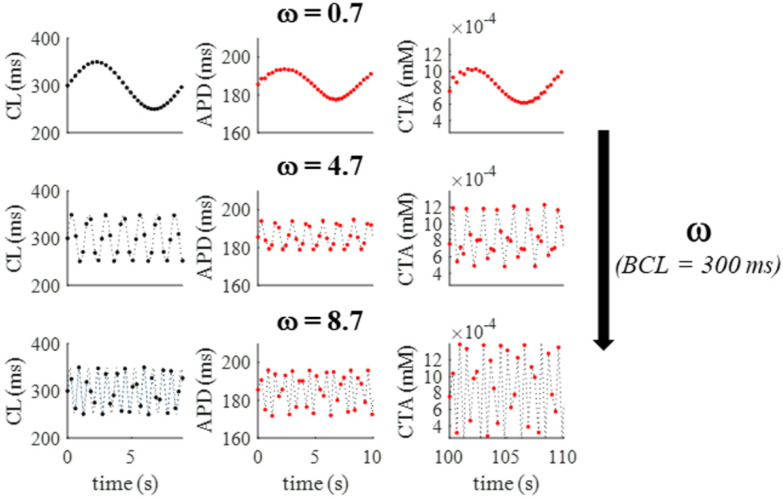
**Dependence of APD and CTA oscillations from ω.** A: When the OR model was paced according to Equation (1) at a high pacing rate (BCL = 300 ms), the amplitude of APD and CTA oscillations increased with ω (black arrow, values reported in figure).

Similarly, when the OR model is paced according to Equation (1) at a fixed pacing CL (BCL = 300 ms), the amplitude of APD and CTA oscillations depends on the rate of beat-to-beat CL changes, i.e., it increases as *ω* does (top to bottom in Figure 3).

**Figure 4:**
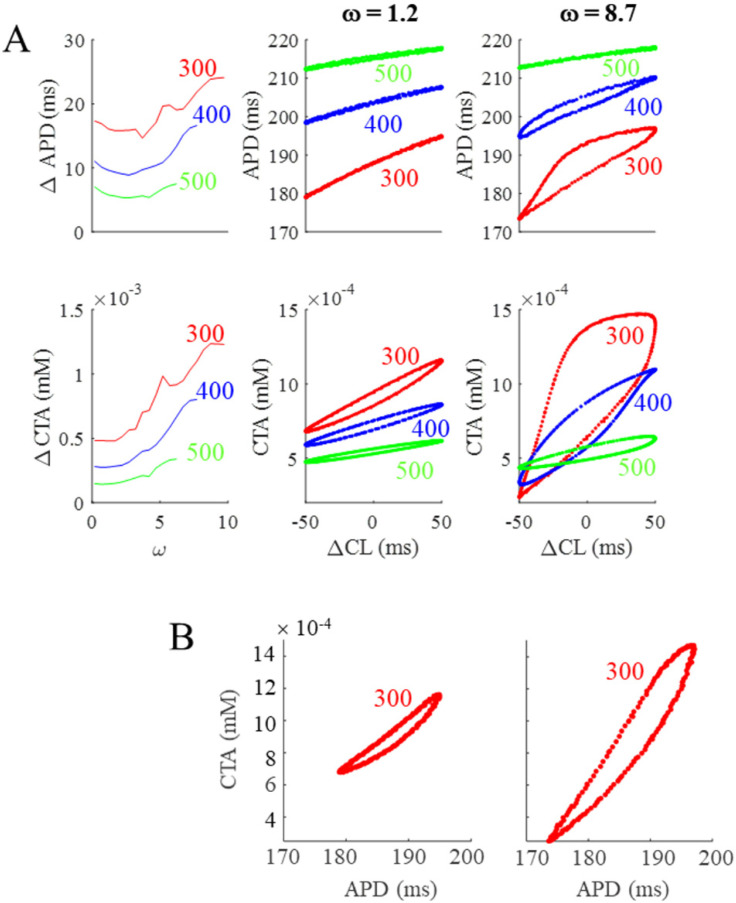
**Summary of BCL and ω dependence of the OR model.** (**A**). First column: ω dependence of APD and CTA oscillation amplitudes (ΔAPD and ΔCTA) under SVP according to Equation (1) at three pacing BCLs (values in ms reported in color). Second column: btb-ER representations of the same APD (top) and CTA (bottom) sequences for a low ω value. Third column: Same for a high ω value. (**B**): Phase plots CTA vs. APD of the same sequences reported in panel A in red. The one at ω = 8.7 is the same also reported in Figure 3C.

The first column of Figure 4 summarizes the results above, by showing that ΔAPD and ΔCTA increase with BCL (300 to 500 ms) and, for each BCL, with ω (0 to 10). The ω range spanned at the three tested BCLs is different for the reasons explained in Methods, being 0–10.5, 0–7.8, and 0–6.3 at BCL = 300, 400, and 500 ms, respectively. In the same figure, the corresponding btb-ER curves for APD and CTA at the three different BCLs are reported for a low (central column) and a high (right column) ω value. The red hysteretic loops in the third column correspond to the simulation reported in the bottom panel of Figure 3 and clearly show that, even at high ω values, and although it does not clearly appear in the corresponding time sequences, APD and CTA do oscillate at the same frequency of CL. This can also be appreciated in the phase plot representations of APD and CTA oscillations (BCL = 300 ms and ω = 8.7) reported in panel B of Figure 4.

### 3.4. Comparison with Other AP Models

**Figure 5:**
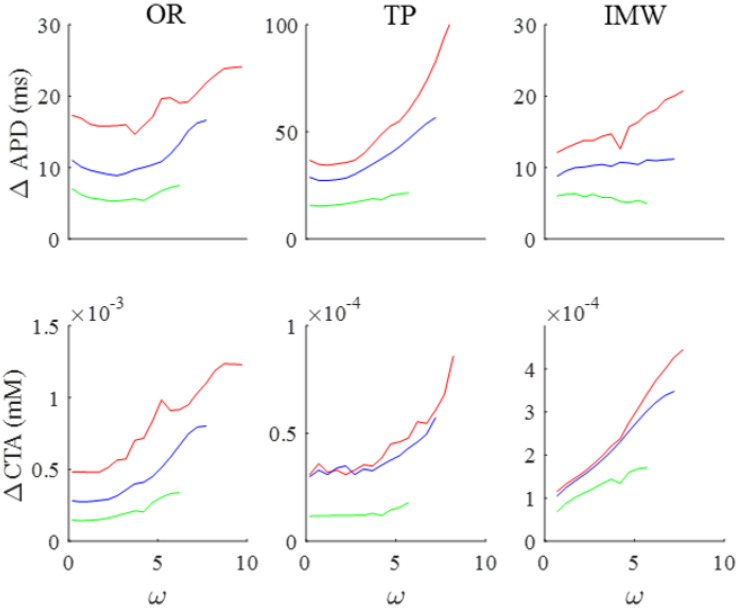
**Comparison of three human ventricular AP models.** Same representation as in the first column of Figure 4 for the OR, TP, and IMW models. Shortest BCL was 300 ms (OR), 350 ms (TP), and 375 ms (IMW), and is reported in red. Intermediate and long BCL were 400 and 500 ms for all models (blue and green, respectively).

The same pacing program applied to the OR model was also tested on TP and IMW human ventricular AP models for comparison. For each model, I used the lowest BCL value, the smallest that did not trigger alternans with ω = 0 (300 ms for OR, 350 for TP, 375 for IMW). The half-interval of CL oscillations σ was 50 ms for OR and TP, and 25 ms for IMW. Despite the differences due to the slightly different rate dependence of the three models, the behavior in terms of amplitude of APD and CTA oscillations under sinusoidal pacing appears qualitatively similar (Figure 5). It is noteworthy that, when paced according to Equation (1) at the shortest pacing BCL, the extent of the increase in the amplitude of the CTA oscillations was always greater (3, 1.6, and 8 times in OR, TP, and IMW) than that of APD. This is also similar for intermediate and long pacing BCL.

### 3.5. The Mass-Spring Model

**Figure 6:**
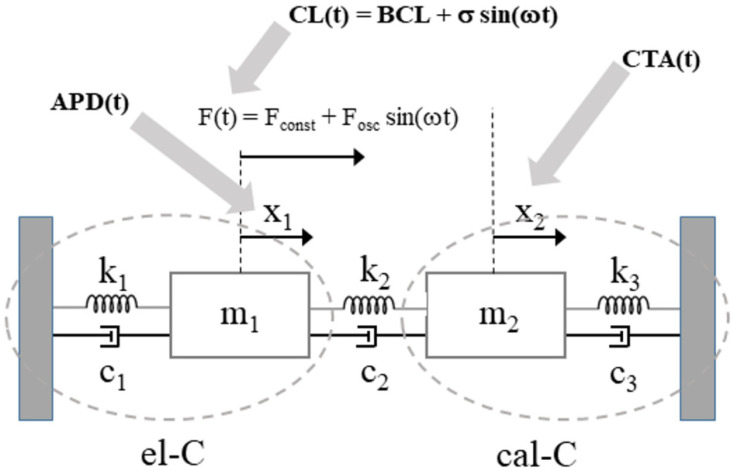
**Schematics of the mechanical model.** The mechanical mass-spring model is made by two masses (m_i_) connected with each other and with fixed walls through springs (stiffness k) and dampers (damping c). Corresponding EC coupling entities indicated by arrows. The system k_1_-c_1_-m_1_ represents the el–C compartment, k_3_-c_3_-m_2_ represents the cal–C compartment, m_1_ is externally driven by a sinusoidal force F following Equation (7) and corresponding to the oscillating CL. Horizontal displacement of the two masses (x_1_ and x_2_) correspond to APD and CTA, respectively. The two oscillators are coupled via spring k_2_ and damper c_2_.

As in the scheme of Figure 1, the cardiac cellular EC coupling can be envisioned as two mechanisms, the membrane electrical excitability (el–C) and the intracellular calcium dynamics (cal–C). The two are bi-directionally coupled through the calcium dependence of SR ryanodine receptors (broken arrow) and that of a number of electrogenic processes underlying excitation (solid arrow). Experimentally, we can follow el–C–cal–C functioning through two of its physiologically significant features, APD and CTA. My above simulations show that when el–C is driven periodically at a given frequency ω, APD and CTA also oscillate with the same frequency (see correlations between APD and CTA in Figure 4B), and the amplitude of oscillations increases as the frequency ω does (Figure 3). The el–C–cal–C machinery behaves similar to a pair of forced coupled oscillators, when the frequency ω of the driving force approaches resonance. Thus, I formulate the hypothesis that the el–C–cal–C dynamics can be modeled with a system of two masses connected reciprocally and with external fixed walls with springs and dampers (MS model), and a periodic force F(t) is applied to only one of the two (Figure 6). To note, the MS model does not mean to simulate the non-linear EC coupling, but rather assume that two relevant observables, APD and CTA, behave, within a broad range of pathophysiological conditions, linearly and, as I show, consistently with the model.

The hypothesis formulated above means that APD and CTA, under oscillatory CL, share the same dynamics (Equation (7)) of the MS model (Figure 6), and I provide here the detailed description of this parallelism. The constant and oscillating components of CL(t) (Equation (1)) control the harmonic behavior of APD(t) and CTA(t), as the constant and periodic components of F(t) (Equation (6)) do on the harmonic dynamics of m_1_ and m_2_ in the MS model. In the former system, the external periodic trigger acts directly to el-C and indirectly, primarily via I_CaL_, to cal–C. Similarly, in the MS model, the external force acts directly on one of the two masses (m_1_). Thus, there is parallelism of the two systems, in which:

1. CL(t) corresponds to the external force F(t) applied to one mass of the MS model.
2. APD and CTA values, and their evolution in time, correspond to the displacement of m_1_ and m_2_ masses along the unique dimension x (x_1_ and x_2_, respectively) in the MS model.
3. The isolated el–C system (uncoupled from cal–C) corresponds to the k_1_, c_1_, and m_1_ system (M_1_S model), where k_1_ and m_1_ determine the normal mode of oscillation (Equation (5)). In this case, CL(t) determines the pace of the current stimulus I_st_.
4. This is the same for cal–C and k_3_, c_3_, and m_2_ (M_2_S model). In this case, when el–C is uncoupled from cal–C, CL(t) determines the pace of I_CaL_.

**Figure 7:**
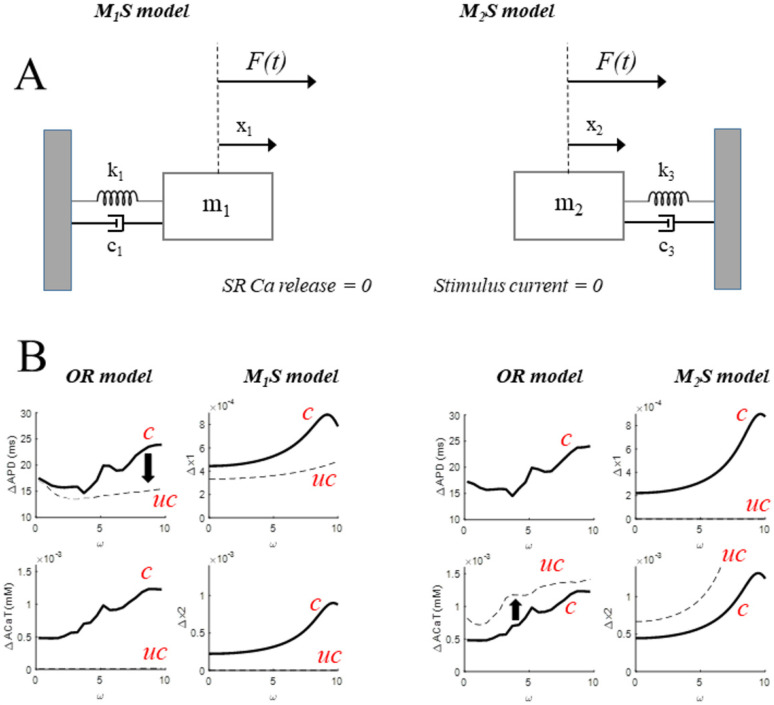
**Comparison between coupled and uncoupled oscillations.** (**A**): When the source of the calcium oscillations is removed by turning SR calcium release off, we hypothesize that the MS model is reduced to a single mass-spring oscillator **(left**). Same in the case of removal of el-C oscillations by turning stimulus current off (**right**). (**B**): The ω dependence of ΔAPD and ΔCTA oscillations at high pacing frequency is reported in first and third columns as a bold line, under SVP following Equation (1) (BCL = 300 ms, σ = 50 ms, 0 < ω < 10). First and third columns show the effects (broken lines) on both parameters of the complete removal of SR release and membrane excitation, respectively, under the same pacing conditions. Second and fourth columns show the behavior of the MS model in coupled (k_2_ and c_2_ ≠ 0, solid line) and uncoupled (k_2_ and c_2_ = 0, solid line) conditions under external sinusoidal forcing. Parameters of the coupled and uncoupled MS model are reported in Table 1. Red labels c and uc stand for coupled and uncoupled state of the two separate oscillators.

Points 3 and 4 assume that el–C and C_c_, when uncoupled and periodically driven, do oscillate at the driving frequency. I test this assumption by driving el–C and cal–C with the sequences of stimuli following Equation (1), while the cal–C and el–C counterparts are, in turn, silenced (Figure 7).

### 3.6. Uncoupling Forced–el–C from cal–C

In the first case, I pace el–C of the OR model with 0.5 ms supra-threshold current pulses delivered according to Equation (1) (BCL = 300 ms, σ = 50 ms, 0 < ω < 10 step 0.2) while setting RyR calcium flux to zero (Figure 7A, left), and representing results similar to Figure 4 and Figure 5 (broken lines in Figure 7B, first column). Although even 100% reduction in RyR calcium-flux preserves small intracellular [Ca^2+^] oscillations affecting, in turn, I_CaL_, I_NaCa_, etc., I consider this the closest condition where el–C is uncoupled from cal–C. The amplitude of the APD oscillations (broken line) at low ω values decreases slightly with respect to the control (solid line) and tends to remain constant over the entire ω range, while the amplitude of the CTA oscillations is reduced to near zero over the same range (bottom panel). The intrinsic resonant frequency of the uncoupled el-C (ω_el-C_), if any, has to be much higher than 10, as it does not produce a significant increase in APD oscillations in the range 0–10. Thus, in order for the M_1_S model (broken lines in Figure 7B, second column) to reproduce the uncoupled–el–C model, its resonance frequency has to be set much higher than 10. This can be achieved by making m_1_ < m_2_ and/or k_1_ > k_2_, without further assumptions on the other MS parameters. The parameters used for the oscillating component of the driving force (F_osc_), for its constant component (F_const_), for the two masses (vector M) and the three stiffness and damping values (vectors K and C) adopted for this simulation are reported in the first column of Table 1. As can be seen in the table, in order to uncouple m_1_ from m_2_, the k_2_ and c_2_ values are simply set to zero. When m_1_ is driven periodically with an external force F1 = F1_const_ + F1_osc_ sin (ωt) (F1, F1_const_, F1_osc_ correspond to CL, BCL, and σ, respectively), the amplitude of the x_1_ oscillations (Δx_1_) does remain fairly constant within the 0–10 ω range (broken line in second column of Figure 7B). Note the difference with respect to the coupled condition (see also Table 1).

### 3.7. Uncoupling Forced–cal–C from el–C

In the second case (Figure 7A, right), I trigger periodic SR calcium releases in the absence of membrane excitation. This is achieved by (1) turning the current injection off in the membrane equation and (2) injecting a constant-waveform I_CaL_(t), previously recorded in control conditions, at a CL following Equation (1) (BCL = 300 ms, σ = 50 ms, 0 < ω < 10 step 0.2). The results in terms of the amplitude of oscillations are shown in the third column of Figure 7B. The amplitude of CTA oscillations (broken line) is always greater than that of the coupled condition (solid line) and increases as ω approaches the maximum value allowed at these pacing conditions (see Methods) at around the value of 10. Since, for ω ~10, the amplitude of the CTA oscillations seems to have reached its maximum value, the value of 10 is taken as the normal mode ω_cal-C_ of cal–C. Of course no APD oscillations are to be seen in el–C, which does not generate APs but only small subthreshold V_m_ oscillations. The M_2_S model endowed with the parameters of Table 1 reproduces the OR behavior for CTA oscillations (Δx_2_) in uncoupled conditions (broken line in the fourth column); coupled conditions are superimposed as solid lines.

### 3.8. The Coupled el–C–cal–C System

Thus, el-C and C_c_ can be separately modeled by means of the externally forced harmonic oscillators of Figure 7A, each one with its intrinsic normal mode of oscillation (ω_el-C_ and ω_Cc_). This allows one to make a guess on the parameters in Table 1 for the two uncoupled condition, where the physical constrain is ω_el-C_ > ω_cal-C_ and ω_cal-C_ ~10. I note that the choice of k and m for M_1_S and M_2_S is really the choice of their ratios k_1_/m_1_ and k_3_/m_2_, which determine the normal modes (Equation (5)). The next step is then to couple the two separate MS oscillators (Figure 7A) into the entire MS model by assigning k_2_ and c_2_ ≠ 0 (Figure 6), and to see if the latter reproduces the physiologically coupled el–C–cal–C system.

The ω dependence of ΔAPD and ΔCTA at BCL = 300 ms (Figure 7B, first and third column, bold solid line) is indeed reproduced by the MS model (Figure 7B, second and third columns, broken solid line).

**Figure 8:**
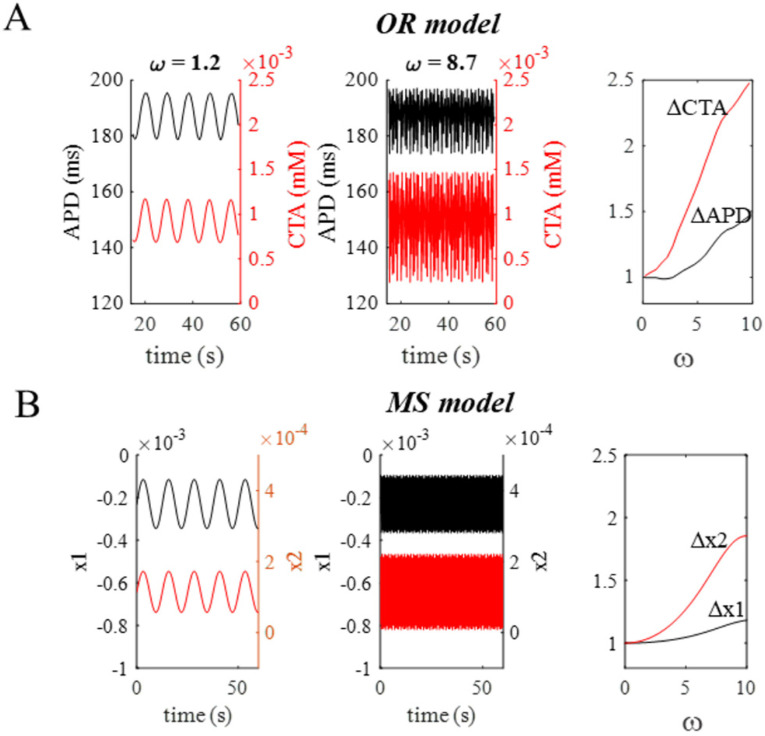
**ω dependence of oscillations in OR and MS models.** (**A**): First panel shows APD (black, left *y*-axis) and CTA (red, right *y*-axis) oscillations of OR model under oscillating CL pacing conditions (BCL = 300 ms, σ = 50 ms, ω = 1.2). Second panel, same at higher ω value. Third panel: amplitude of APD and CTA oscillations normalized to their value at ω → 0, as a function of ω. (**B**): Same representation as in A, for x1 and x2 of the MS model (parameters in Table 1).

A detailed example of APD (black) and CTA (red) oscillations when the OR model is electrically paced with oscillatory CL (BCL = 300 ms, σ = 50 ms) at a low (first panel) and a high (second panel) ω values is reported in Figure 8A. ΔAPD and ΔCTA are shown as functions of ω in the third panel, by assuming, for the sake of comparison, Δvalues = 1 at ω → 0. When ω increases, both the amplitudes of the APD and CTA oscillations increase as well (from 17.5 to 22.5 ms the former, and from 0.5 × 10^−3^ to 1.2 × 10^−3^ mM the latter), while their average values do not change (190 ms and 0.9 × 10^−3^ mM, respectively). Panel B shows the response for the same ω values of the MS model, with parameters of Table 1 except for a greater k_1_ value of 795. The MS model with these parameters, which will be kept for the rest of the study, retains the features described in the previous section.

### 3.9. Pacing-Rate and Beat-to-Beat-Rate Dependence of APD and CTA Oscillations

The first of the points that I have listed above on the parallelism between EC coupling and MS model is the correspondence of CL(t) with the external force F(t) (Equations (1) and (6)). The ω dependence of oscillation amplitudes has been shown in the previous paragraphs in the case of BCL = 300 ms (see Table 1). The correspondence also holds between the constant component BCL and F_const_. When ω = 0, in fact, the cell membrane is triggered with a constant CL(t) = BCL, and APD and CTA assume their steady state rate-dependent values, which would result for instance in flat APD and CTA curves (185 ms and 8 × 10^−4^ mM, respectively) in the example of Figure 3. This is the case in the MS model, where a constant force is only applied to m_1_, and both m_1_ and m_2_ are displaced to constant x_1_ and x_2_ values.

**Figure 9:**
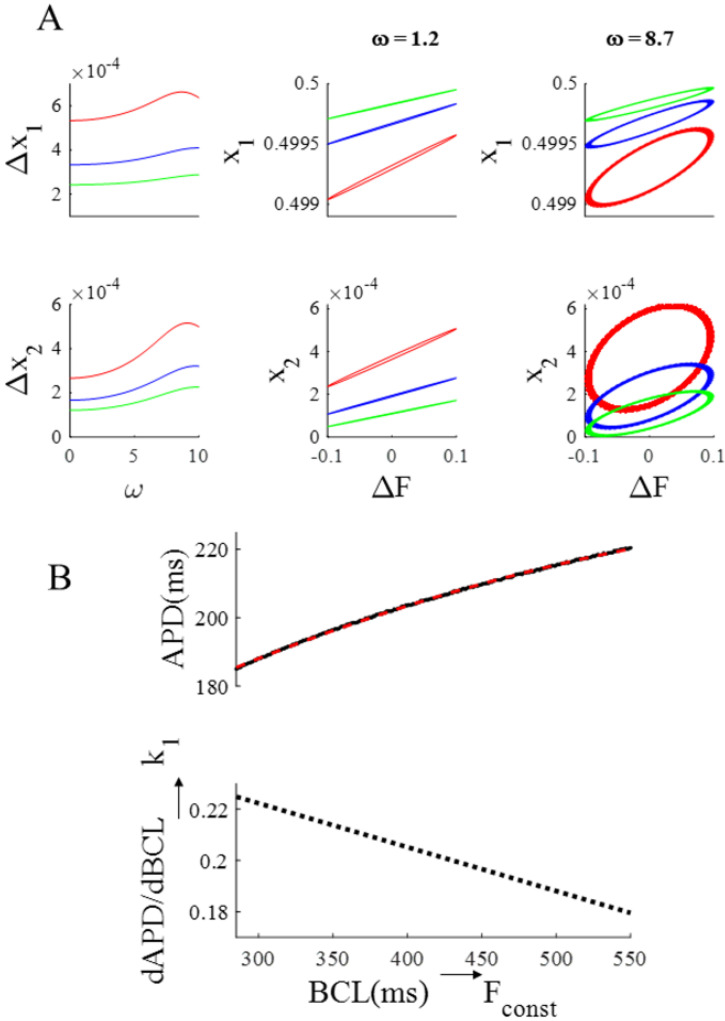
**Summary of F_const_ and ω dependence of MS model.** (**A**): Same as Figure 4, where the external force F (Equation (7)) takes the place of CL (Equation (1)), F_const_ of BCL, F_osc_ of σ, x_1_, and x_2_ of APD and CTA, and ΔF of ΔCL. F_const_ was assigned in turn with 3 different values (0.26, 0.2, and 0.14, respectively, reported in red, blue, and green), and k_1_ made linearly dependent on F_const_ (see Table 2). (**B**): Top, steady state rate dependence curve for APD of the OR model for the BCL range under study (black). The curve was fitted (R = 0.99) with a quadratic (red) a × BCL^2^ + b × BCL+c (a = −0.000171; b = 0.2736; c = 121.4), whose linearly decreasing slope is reported in the bottom panel.

In order to fit the BCL dependence (different colors) of OR results in Figure 4, the stiffness k_1_ of the MS model had to be made linearly dependent on the constant component of the force driving m_1_ (F1_const_) (Table 2). In other words, k_1_ decreases as the constant tension F_const_ on m_1_ (corresponding to BCL) increases and makes the MS model reproducing both BCL and ω dependence of APD and CTA (Figure 4 and Figure 9A). The steady state rate dependence of APD in the BCL range under study is fitted (R = 0.99) by a quadratic curve (black and red in top panel of Figure 9B), whose slope therefore decreases linearly in the same CL range (bottom panel). We can therefore directly associate (apart from a scale factor) the slope of rate dependence with the stiffness of the el–C oscillator.

Thus modified, the MS model also reproduces the fact that, shown in Figure 4 and specifically discussed in previous works [22,24,25], as both BCL and/or ω increase, the btb-ER representations (APD vs. CL and CTA vs. CL) assume a hysteretic form. In other words, as the frequency of the oscillation of APD and CTA is the same as that of pacing CL, a phase difference develops depending on whether beat-to-beat ΔCL is positive or negative. I will refer to this fact, for brevity, as “phase behavior” in the following discussion.

**Figure 10:**
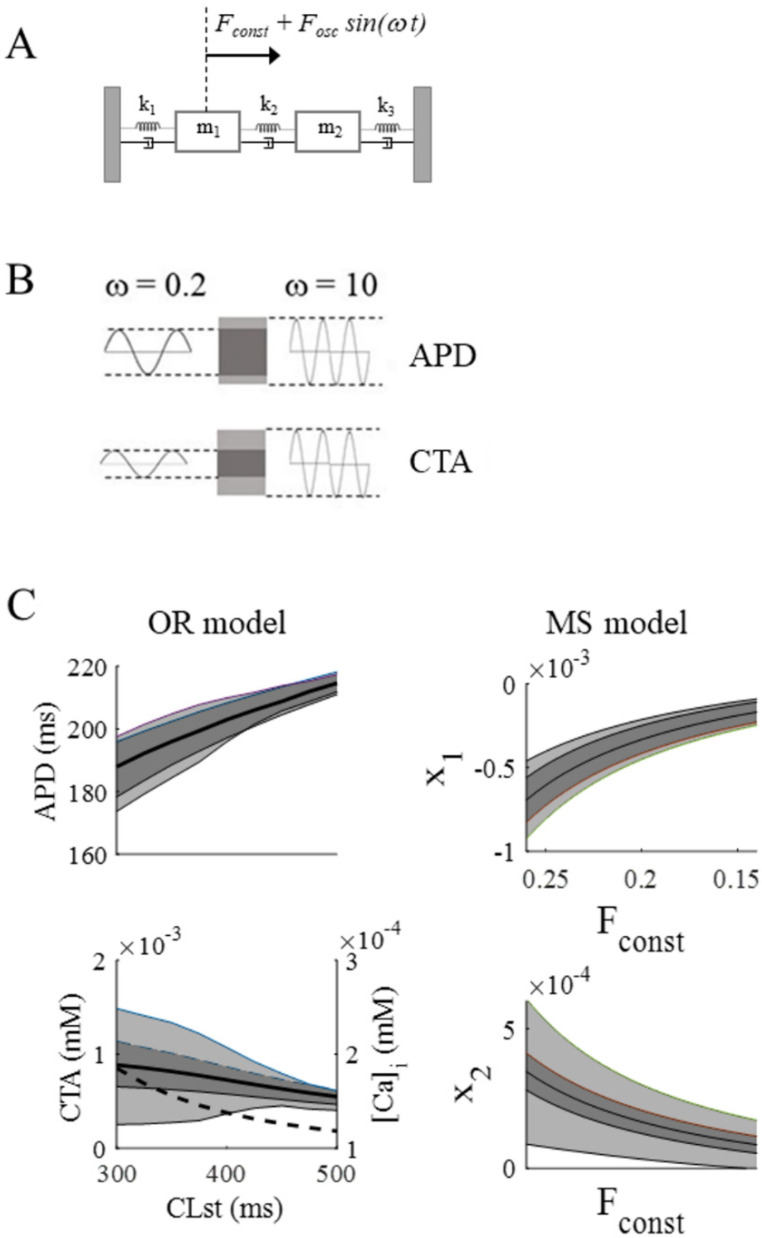
**MS model reproduces both rate dependence and ω dependence of oscillations.** The mechanical model, modified by making the stiffness k_1_ linearly dependent on F_const_ (panel (**A**)), was forced according to Equation (7), with 0.14 < F_const_ < 0.26, at low (0.2) and high (10) ω values, while the OR model was paced according to Equation (1) at the same ω values. Results of simulations at low and high ω are reported as dark and light grey regions, respectively (scheme in panel (**B**)). Left column of panel (**C**) shows the average value (black line) and the oscillation amplitude of APD (top) and CTA (bottom) as explained in panel B, for BCL ranging from 300 to 500 ms. Superimposed in the bottom panel (broken line), the end-diastolic value of intracellular calcium concentration. On the right column, x_1_ and x_2_ oscillations when the MS model was forced as explained above.

Rate and ω dependence of OR model oscillations are further summarized in the first column of Figure 10B, where BCLs from 300 ms to 500 ms step 20 ms, σ = 50 ms, and 2 ω values were simulated. The scheme in panel A explains the way APD and CTA oscillation amplitudes are reported in panel B, for small (dark grey) and large (light grey) ω values. The solid black curves in panel B represent APD and CTA steady state rate dependence. The dotted curve in the lower panel represents end-diastolic calcium concentration (right Y axis). The second column of panel B reports MS model simulations, where F corresponds to CL (F_const_ to BCL, and F_osc_ to σ). Thus, with decreasing pacing rate (F_const_), APD (x_1_ displacement) increases and CTA (x_2_ displacement) decreases, whereas the amplitude of their oscillations for large excursions of beat-to-beat CL (F) variability slightly decreases in both instances.

### 3.10. Exploring Harmonic Features of EC Coupling

**Figure 11:**
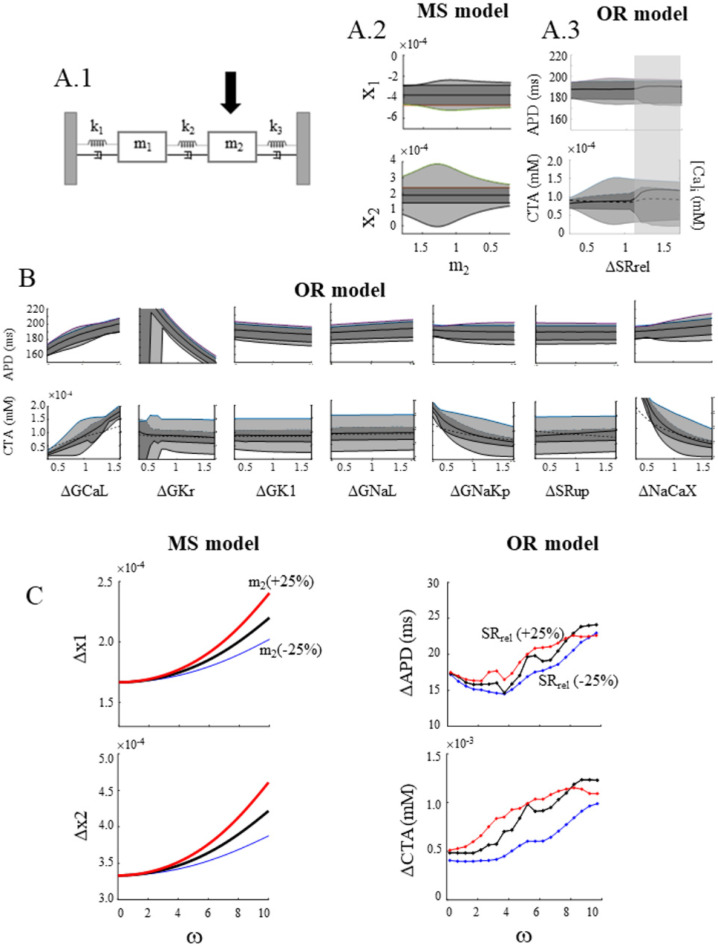
**SR calcium release and inertia of cal**–**C compartment.** The same representation of Figure 10 is provided for x_1_ and x_2_ oscillation amplitudes when m_2_ increases or decreases (panels (**A1**) and (**A2**)). The same behavior is observed in oscillation amplitudes of APD and CTA of the OR model when SR calcium release undergoes ±50% changes (panel (**A3**)). Analogous representation was measured in the OR model for changes in a number of maximum conductance of ion currents and membrane transporters (panel (**B**)). Panel (**C**), left column shows the left (right)shift of the resonant profile of the MS model for x_1_ and x_2_ oscillation amplitudes when m_2_ was increased (decreased) by 25%. Panel C, right shows the left (right) shift of the ω dependence of APD and CTA oscillation amplitudes (BCL = 300 ms) when the flux through SR release channels was increased (decreased) by 25%.

A first attempt to explore specific events of cellular EC coupling in terms of coupled harmonic oscillators is shown in Figure 11. Here, I assume the harmonic hypothesis presented above and wonder which component of the EC coupling machinery plays the role of the inertia m_2_ of the cal–C oscillator. I will use representations here that are similar to those in Figure 10 since they summarize the differential effect of changes in one parameter (reported in the *x*-axis) on the average value and amplitude of APD and CTA oscillations at low (dark grey) and high (light grey) ω values.

Panel A.2 summarizes the expected changes in Δx_1_ and Δx_2_ when m_2_ of the MS model (panel A.1) is increased or decreased around its control value (Table 2). When I derive the same representation by testing, in the OR model, ±50% changes in a number of ion currents, transporters, and calcium fluxes (panels A3 and B), I find that the one that more closely reproduces m_2_ changes is that corresponding to the modulation of the SR calcium flux to the cytoplasm. The shaded area in panel A3 represents conditions where CTA alternans develops in the OR model and cannot find its counterpart in the linear MS model. I then recalculate the ω dependence of ΔAPD and ΔCTA for the OR model (black curves in panel C, right column), and I find that a ±25% modulation of the total SR calcium flux leads to left/right displacement of the resonant profile (red and blue, respectively), as predicted from the same changes in m_2_ of the MS model (panel C, left column).

**Figure 12:**
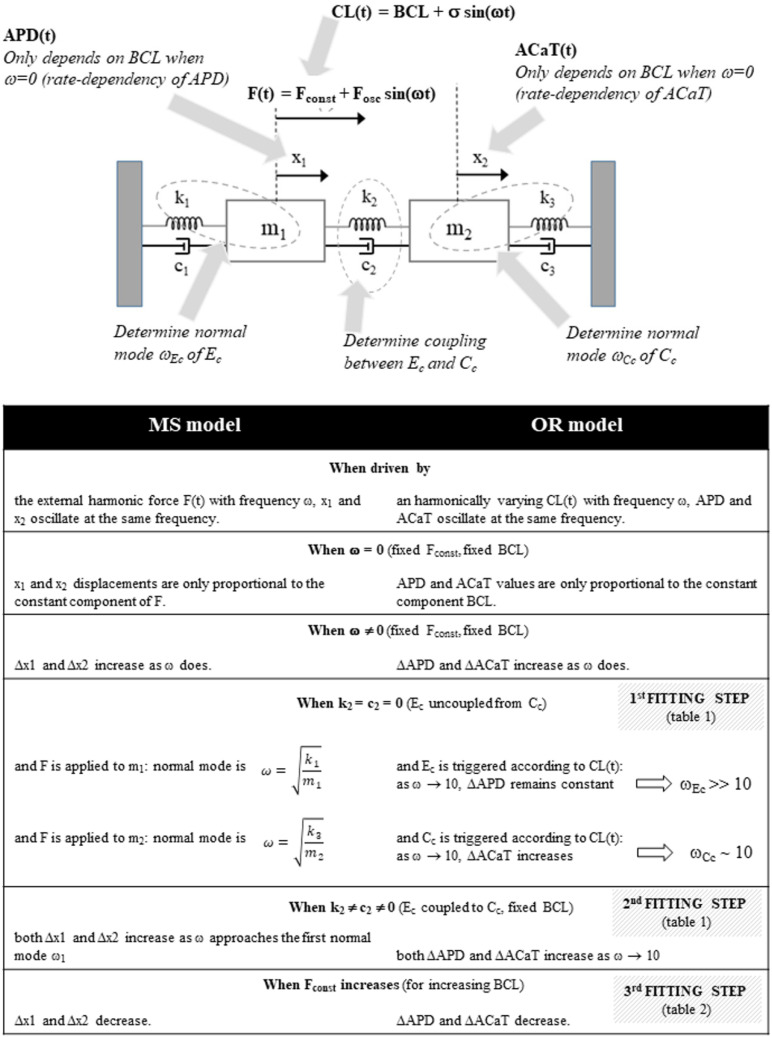
**Parallelism between OR and MS models.** (**Top**): The MS model with schematic reminds to the corresponding EC coupling players. (**Bottom**): Schematic features of the parallelism between MS and OR behavior under SVP.

The main facts on which the parallelism between the OR system and MS model is based are summarized in the schematic Figure 12, where the three main steps in the fitting procedure of the MS model’s parameters with OR results are also synthetically reported.

## 4. Discussion

We know that, even at a beat-to-beat basis, a shortening of pacing CL leads to a shortening of ventricular APD, which in turn leads to a decrease in CTA. This implies that under variable pacing rate, the time course of CL, APD, and CTA correlate in phase, i.e., by driving membrane excitability sinusoidally, APD and CTA oscillate at the same frequency of the pacing CL, as coupled harmonic oscillators do. From a dynamic point of view, harmonicity becomes interesting if some resonant behavior emerges under common operating frequencies of the system. Therefore, by moving from this analogy, this study aims to (i) investigate whether ventricular repolarization and calcium cycling resonate with an oscillating pacing CL, and (ii) discuss the possibility of using harmonic oscillator theory to model their dynamics. Fitting procedures for both APD (or CTA) rate dependence and restitution have been proposed. Perhaps the better established is that of Elharrar and Surawicz [26], with a hyperbolic relationship between constant pacing CL and steady state APD values (rate dependence), which are reached over consecutive beats according to a bi-exponential law (restitution). Besides mechanistic explanations of the involved fitting parameters, which have been given over the years [27], these approaches have been invaluable for understanding and predicting cardiac dynamics from the cellular to the clinical level. Although synthetic pictures of the interplay between rate-dependent and restitution properties have been studied [28], ideally it would be desirable to have a unique dynamical law encompassing both. (iii) This constitutes the third aim of the present study.

Cardiac physiologists have been interested in the way APD and CTA, key determinants of the contraction force developed by the heart, sense steady state or sudden changes in pacing CL, respectively, denominated rate dependence and restitution. Rate dependence, for a given cell, is unique, in the sense that APD and CTA values are defined once and for all CLs, whereas there are infinite restitution curves, one for each pacing CL [24]. The restitution hypothesis suggests that a restitution slope greater than 1 predicts an increased instability of AP repolarization and is frequently associated with increased risk of arrhythmia development [29,30]. Despite its relevance in theoretical, experimental, and clinical research, the hypothesis suffers from two limitations: first, it is defined for sudden and single pre/post-mature perturbations of a constant CL [8], whereas pacing rate is always subject to a certain degree of beat-to-beat variability [31]. Second, a growing number of experimental findings violate the hypothesis, by showing APD and/or CTA alternans with a restitution slope smaller than 1, or no alternans with a slope greater than 1 [9,32,33,34]. A key point is that both APD and CTA respond to sudden changes in pacing rate with their restitution dynamics, though it is still not clear which one leads the other under increased variability of pacing rate [17,34,35,36]. Several hypotheses have been formulated in order to explain these facts [11,37], also by showing that intracellular calcium cycling can per se constitute a source of alternans [11,35,38]. However, the way repolarization and calcium dynamics affect each other is extremely complex and multivariate, and a theoretical framework to separate their contribution to heart rate variability is still lacking.

Together with progressively and biologically more complex numerical reconstructions [39], there seems to be a need for abstract models that can synthetically capture key aspects of EC coupling dynamics.

### 4.1. Why a Mass-Spring Model

The instances in which harmonic oscillator theory is adopted to explain and control natural phenomena can hardly be enumerated, encompassing fields such as electronics, mechanics, construction engineering, and physiology. Among the advantages of this theory is the possibility of defining the natural frequency of an oscillator, most of the times in order to avoid it. In fact, when a harmonic oscillator is driven with an external frequency approaching its natural one, resonance occurs, and oscillations tend to increase in magnitude, potentially leading to structural or functional damage [40]. The typical harmonic oscillator model is the mass-spring system reported in Figure 7A. According to the theory, we can force *m*_1_ to oscillate under a sinusoidal *F(ω,t)* and avoid, for example, resonance, by making ω << ω_1_, either by increasing the stiffness *k*_1_ or decreasing the mass *m*_1_ (Equation (5)). The value of the damping constant *c*_1_ does not modify the normal mode, but only the amplitude and the range of the resonant response. When applied to N mechanically coupled oscillators, the theory predicts their N normal modes and their increasingly more complex dynamics. The case of N = 2 is adopted here to simulate the dynamics of the el–C–cal–C system when externally driven by a sinusoidally oscillating CL. It should be pointed out that there is no physical reason for the particular choice of a mechanical model. An electrical inductance–capacitance circuit (LC oscillator), or any other linear harmonic oscillator would have served the scope as well. Similarly, there is no physical connection between the mechanical parameters and the electrophysiological ones, other than they share the same dynamics described by Equation (7).

### 4.2. Bi-Directional Coupling between AP and CT

The main relevant result of the present study is that a resonant-type behavior is indeed observed between simulated APDs/CTAs and oscillating CLs, and the resonant frequency of el–C (ω_el-C_) appears to be much higher than that of cal–C (ω_cal-C_) (Figure 7B) when both are driven with the same CL(t). OR results are qualitatively well fitted by MS model parameters consistent, as uncoupled simulations require, with an ω_el-C_ >> 10 and an ω_Cc_ ~10 (17.3 and 8.7, respectively, see Table 1 and Figure 7).

According to the theory, the lowest one between the normal modes of M_1_S and M_2_S sets the lower limit for the two normal modes of the coupled MS system. Parameters in Table 1 demonstrate the working hypothesis: EC coupling can be conceived, in its APD and CTA state variables, as a system of two coupled harmonic oscillators, where only the cal–C oscillator, when isolated, resonates in the ω-ROI, and brings therefore the major contribution to the resonance of both el–C and cal–C observed in physiological conditions (Figure 7B). More accurate fitting procedures can be thought of, as the two values for ω_el-C_ and ω_Cc_ have been basically hand-tuned through the corresponding *k* and *m* constants. I note however that because the linearity of the solutions of the harmonic oscillator and the fact that resonance frequency only depends on these two parameters, better fitting accuracy would not dramatically change MS model results.

It is therefore the way calcium cycling senses CL variability that chiefly determines the way this variability affects repolarization, and not vice versa. Sato and co-workers have found that calcium instability leads to APD instability [11,35] in conditions where the former is increased: CT-alternans slaves APD, in their words. What the MS model suggests is that the leading role of calcium dynamics over electrical dynamics is not limited on the “who moves first” in unstable conditions, but rather appears to be an intrinsic property of cellular EC coupling for a broad range of pacing conditions, including physiological ones.

The linear Newton’s equations system of the MS model (Equation (7)) can be viewed as a transfer function acting as a filter and transforming a series of beat-to-beat varying pacing CLs into a series of APDs and CTAs. The characteristic Equation (11) also provides the resonant modes of the electrical and calcium compartments. More complex filtering functions have been proposed previously [15,41].

### 4.3. Resonance in the Cardiovascular System

I have shown that the normal oscillating mode of cal–C which appears within physiological pacing conditions (300–500 ms for BCL, 0–50 ms for σ) is lower than that of el–C and is accessible experimentally. Other resonant phenomena within the cardiovascular system have been found. Again, what is relevant is not the existence of a theoretical resonance in a cyclic system, but the fact that it can be measured and corresponds to working conditions which are relevant for its pathophysiology.

Rubenstein and co-workers measured the intrinsic frequency of spontaneous SR oscillations in CT right ventricular myocytes and noted that periodic stimuli can entrain SR oscillations in a harmonic fashion at CLs different from the intrinsic one [42]. SR intrinsic calcium oscillations have also been measured in rat ventricular myocytes in the range 0.3–3 Hz [43,44].

A major difference with respect to my work is that these studies assume resonant calcium frequency as the one reached during spontaneous calcium oscillations after overloading the SR. Calcium overload severely modifies the SR dynamics, whereas the external periodic I_CaL_ I use to trigger cal–C explores the resonance of a physiological SR. The second difference is that they study the non-linear dynamics of membrane potential and calcium release, whereas I focus on the dynamics of the relatively linear APD and CTA observables, i.e., the reaching of the excitation threshold and the appearance of SR release are not variables in my simulations.

Resonance at approximately 0.1 Hz has also been reported in heart rate variability (HRV) mainly in response to breathing. When respiration rate approaches resonance, it leads to greater amplitude oscillations in heart rate, blood pressure, and vascular tone [45,46]. Other sources of rhythmic stimulation, such as oscillations in the autonomic input to the heart and rhythmic muscle tension, have been proposed to take part in what has been defined HRV biofeedback resonance [47]. Arterial baroreflex is likely to modulate this effect [48] and also periodic changes in membrane ion currents, in turn driven by oscillations of the energy metabolism at a rate of approximately 0.1 Hz [49].

### 4.4. Oscillatory Features of EC Coupling

A finer tuning of the MS model’s parameters goes beyond the scope of this paper, which aims to primarily demonstrate that the conditions of resonance exist, can be measured, and can be used to model EC coupling dynamics. Nevertheless, even in its present simple form, the MS model can be used to investigate dynamic aspects of cardiac EC coupling in detail. As explained in the results section, the simulations of Figure 11 show that either the SR calcium content or the peak of CT are chief determinants of the cal–C inertia and, through that, of the increase in the amplitude of both APD and CTA oscillations under oscillating CL. Based on the MS model’s parallelism, we can speculate that the compartment leading beat-to-beat variability of EC coupling is the calcium one (Figure 7). Following the parallelism, E_c_’s sensitivity to ω changes in SVP is mainly controlled by the SR release, the same way the resonance of the MS model is determined by the inertia of the second mass *m*_2_ (Figure 11C). As noted above, it has long been debated whether APD and CTA alternans are electrically or calcium driven, particularly in cases where pathological, pharmacological, or genetically inherited alterations of calcium-dependent ion channels are involved [50]. The theoretical and experimental difficulty in this controversy is inherent to the complexity of EC coupling, where two non-linear cycles, the electrical and the calcium ones, are coupled by a mechanism affecting both. The MS model shows, on the other hand, that the two observables APD and CTA behave, within a broad range of pacing conditions, linearly and, indeed, harmonically. Though the above explanation might still be speculative, I think that it can draw new light into this long-standing debate.

It should be noted in addition that all the above results hold not only for changes in the beat-to-beat (ω) component of CL, but also for changes in its constant component BCL, as a result of making the stiffness *k*_1_ of the M_1_S linearly dependent on the constant component of the external force. This accounts for the resonant effect (the ω dependence of APD and CTA oscillations) becoming more evident at higher pacing rates (see first column of Figure 4A and Figure 9A), and is consistent with BCL exerting a direct effect only on the dynamics of the electrical compartment el–C. From a physiological point of view, the rigidity *k*_1_ of the el–C compartment corresponds to the slope of the steady state rate dependence curve of APD (Figure 9B), thus providing a direct tool to experimentally assess this parameter. The slope of this curve, particularly at low BCL values, has been shown in isolated ventricular myocytes to be chiefly dependent on the maximum conductance of the inward rectifier potassium current I_K1_, with some contribution from the inactivation kinetics of the rapid delayed rectifier I_Kr_ [27]. This provides a starting point for further exploration of the electrophysiological mechanisms controlling the stiffness of the el–C oscillator.

In the present study, I have deliberately avoided considering pacing BCL values lower than 300 where human ventricular myocytes in general, and the OR model in particular, display bi-stability in the curve of APD rate dependence [16,22], which results in APD alternans. I note however that bi-stable elastic elements have been recently shown to introduce, in coupled mass-spring systems, non-linear dynamics that turn on and off according to the amplitude of oscillatory driving force [51]. It is not unlikely therefore that the parallelism between the slope of APD rate dependence to the stiffness of the electrical oscillator could hold also for studying transitions from stable to unstable repolarization and calcium dynamics conditions such as those of APD and CTA alternans that frequently precede cardiac arrhythmias.

Similarly, I have found a striking similarity between the modulation of maximum conductance of I_CaL_ in the OR model (first panel of Figure 11B) and that of *k*_2_ in the MS model (not shown), which is not surprising as the L-type calcium current is mainly responsible for coupling el–C and cal–C compartments. This is consistent with the finding that the calcium current is not affected during alternans, which is rather caused by changes in the gain of EC coupling [52,53], i.e., changes in the calcium current do not modify the dynamics of cal–C but only control the extent of its coupling to el–C.

### 4.5. Possible Developments of the Mechanical Framework

The physics of harmonic oscillators can retain its simplicity even when faced with high degrees of freedom and with structural complexities in the model design. In this sense, the harmonic model of cardiac EC coupling, when confirmed through in vivo experiments, can become a first brick in a larger construction. The source of EC coupling results of this study is the OR model of human ventricular non-propagated AP, but findings are consistent in two additional models (Figure 5). The approach can indeed be suitable for any numerical model of the cardiac AP and it can be directly conceived for patch clamp studies on isolated cardiomyocytes. More complex MS models can be thought of based on EC coupling details not included in the OR reconstruction, as well as by extending the harmonic model to the whole organ physiology anytime sinusoidal-type pacing is present or can be introduced.

For example, calcium–contraction coupling can be included in a three masses four springs model, by adding a contraction compartment to the MS model, which would allow one to consider mechanoelectrical interactions as part of the oscillating dynamics.

Finally, the possibility, yet to be proved, of fitting, according to a harmonic model, the relationship between QT variability of surface ECGs and that of intra-ventricular or arterial blood pressure under RR variable pacing, would allow the non-invasive estimate of functional parameters, such as the extent of electromechanical coupling, from clinical measurements.

### 4.6. Study Limitations

A limitation of this study is that most of the presented results only qualitatively describe the oscillating properties of numerical AP models in terms of the MS model’s parameters, as my primary goal was to demonstrate the consistency of the harmonic model hypothesis. More systematic fitting procedures need to be undertaken in order to better describe the complexity of EC coupling details. Additionally, the role of damping constants is only touched in the present account (Figure 9B) and their dynamic involvement still has to be addressed. Finally, findings need to be confirmed in vivo. Nevertheless, a preliminary validation of the harmonic hypothesis on numerical models provides a solid starting ground. Hodgkin–Huxley-type equation systems, such as the OR, compactly summarize our up-to-date knowledge of the EC coupling dynamics and allow the controlling and monitoring of all the variables in play, and the amount and complexity of experiments involved at this stage was hardly conceivable for an in vivo approach.

## 5. Conclusions

In summary, SVP protocols reveal:

1. A resonant behavior of cellular APD and CTA with pacing CL.
2. The possibility of assessing normal modes of the two observables and including their steady state and beat-to-beat dynamics into a unique law.
3. The normal mode of CTA oscillation falls within the physiological range of pacing variability and appears to be the one leading the increase in APD oscillations when variability increases, particularly at a high pacing rate.
4. A preliminary parallelism is also documented between the slope of steady state APD rate dependence and the stiffness of the el–C compartment, as well as between the SR calcium release and the inertia of the cal–C compartment.

These observations can have immediate application in the growing field of numerical reconstruction of cardiac cellular action potentials [39], as well as in single cell studies of cardiac electromechanics. Significant future implications can be expected in the non-invasive dissection of whole-heart dynamical parameters, relevant for the clinics and therapy of cardiac rhythm disorders.

## Figures and Tables

**Table 1:** **Parameters of the MS model.** Model’s parameters in the case of M_1_S (Figure 7A left), M_2_S (Figure 7A right), and MS (Figure 7C). Oscillating (F1_osc_, F2_osc_) and constant (F1_const_, F2_const_) components of the external force driving mass 1 (m_1_) and mass 2 (m_2_), vector of the two masses (M), vector of the three stiffness constants (K), vector of the three damping constants (C), and resonant frequencies of the three systems.

	M_1_S Model	M_2_S Model	MS Model
**F1_osc_**	0.1	0.0	0.1
**F1_const_**	0.2	0.0	0.2
**F2_osc_**	0.0	0.1	0.0
**F2_const_**	0.0	0.2	0.0
**M**	1, 2	1, 2	1, 2
**K**	300, 0, 150	300, 0, 150	150, 150, 150
**C**	5, 0, 5	5, 0, 5	5, 5, 5
**ω_res_**	17.3	8.7	8.7, 12.2

**Table 2:** **Parameters of the MS model.** Parameters as explained in Table 1. The stiffness constant k_1_ depends linearly on F1_const_, with a = 1275 and b = 3750.

	MS Model
**F1_osc_**	0.1
**F1_const_**	from 0.14 to 0.26, step 0.01
**F2_osc_**	0.0
**F2_const_**	0.0
**M**	1, 2
**K**	a – b × F1_const_, 150, 150
**C**	5, 5, 5

## Symbols and Abbreviations

APD	action potential duration, as measured at V_m_ = −60 mV
CTA	amplitude of the calcium transient as measured between diastolic and peak value
BCL	basic pacing cycle length
btb-ER	beat-to-beat electrical restitution curve of AP sequence (every APD vs. preceding CL)
cal–C	calcium compartment of the EC coupling
CL	pacing cycle length
*c*_1_, *c*_2_	damping constants of the MS model
*m*_1_, *m*_2_	masses of the MS model
*k*_1_, *k*_2_, *k*_3_	stiffness constants of the MS model
el–C	electric compartment of the EC coupling
F	total external force applied to the MS model
F_const_	constant component of F
F_osc_	oscillating component of F
MS model	two-degrees-of-freedom mass-spring model
M_1_S model	isolated (from *m*_2_) left harmonic oscillator
M_2_S model	isolated (from *m*_1_) right harmonic oscillator
OR model	numerical model of human ventricular AP (16)
SVP	sinusoidally varying pacing protocol
ω	angular frequency of sinusoidal CL oscillations to OR model, and of external force to MS model
ω−ΡOΙ	range of physiological interest for ω
